# Problem-Based Training Improves Recognition of Patient Hazards by Advanced Medical Students during Chart Review: A Randomized Controlled Crossover Study

**DOI:** 10.1371/journal.pone.0089198

**Published:** 2014-02-20

**Authors:** Friederike Holderried, Daniel Heine, Robert Wagner, Moritz Mahling, Yelena Fenik, Anne Herrmann-Werner, Reimer Riessen, Peter Weyrich, Stephan Zipfel, Nora Celebi

**Affiliations:** 1 University of Tuebingen, Medical School, Office of the Dean of Student Affairs, Tuebingen, Germany; 2 University Hospital of Tuebingen, Department for Anaesthesiology, Tuebingen, Germany; 3 University of Tuebingen, Medical School, Tuebingen, Germany; 4 University Hospital of Tuebingen, Department for Internal Medicine VI, Psychosomatic Medicine, Tuebingen, Germany; 5 University Hospital of Tuebingen, Medical Intensive Care Unit, Tuebingen, Germany; 6 University Hospital of Tuebingen, Department for Internal Medicine IV, Angiology, Endocrinology, Nephrology and Clinical Chemistry, Tuebingen, Germany; 7 Nephrology Center Marienpark, Stuttgart, Germany; Iran University of Medical Sciences, Iran (Republic of Islamic)

## Abstract

**Background:**

Patient chart review is the gold standard for detection of potential patient hazards (i.e. medication errors or failure to follow up actionable results) in both routine clinical care and patient safety research. However, advanced medical students’ ability to read patient charts and to identify patient hazards is rather poor. We therefore investigated whether it is possible to teach advanced medical students how to identify patient hazards independent of context (i.e. cancer versus cardiac failure) in patient charts.

**Methods:**

All fifth-year medical students in one semester (n = 123) were randomized into two groups. One group (IC) received a patient chart review-training first and then a control-intervention and the other group (CI) received the control-intervention first and then the patient chart review-training. Before and after the teaching sessions, students reviewed different scenarios with standardized fictional patient charts containing 12 common patient hazards. Two blinded raters rated the students’ notes for any patient hazard addressed in the notes using a checklist. The students were blinded to the study question and design. There was no external funding and no harm for the participating students.

**Results:**

A total of 35 data sets had to be excluded because of missing data. Overall, the students identified 17% (IQR 8–29%) of the patient hazards before the training and 56% (IQR 41–66%) of the patient hazards after the training. At the second assessment students identified more patient hazards than at the first. They identified even more in the third. The effect was most pronounced after the patient chart review training (all p<.01).

**Conclusion:**

Patient chart review exercises and problem-based patient chart review training improve students’ abilities to recognize patient hazards independent of context during patient chart review.

## Introduction

Some common patterns constitute a large fraction of patient hazards. According to the data of the National Patient Safety Benchmarking Center, five most frequent medical errors and adverse events (surgical errors, medication errors, medical errors, patient falls, nosocomial infections) account for 67% of medical errors and for 81% of the costs attributable to medical errors [1]. In internal medicine, medication problems and diagnostic errors are frequent patient hazards [2].

Patient chart review is considered to be the gold standard for the identification of many common patient hazards, especially diagnostic problems (delayed, missed, wrong diagnosis, failure to follow up on actionable test results), medication problems (overmedication, undermedication, wrong dosage, medication contraindications, unauthorized medication, drug-drug interactions), nosocomial infections and problematic fluid/diet-management [3], [4], [5], [6], [7], [8], [9]. In patient safety research, patient charts are retrospectively reviewed by a nurse or physician specifically trained for this task [10].

Since patient chart review should be routinely performed during every ward round, it has the potential to be a major tool for the promotion of patient safety. Previous investigations have shown that advanced medical students’ ability to read and interpret patient charts is rather poor: Nikendei et al. performed ward round exercises with final year medical students and found that the students performed only 49% of the expected tasks during patient chart review, prescribing and documentation [11]. In a previous study by our group, 5^th^ year medical students identified only 20% of the patient hazards in fictional patient charts, irrespective of the number of weeks they spent in clerkships in internal medicine [12]. In a study by Heaton, which included 2413 medical students and recently graduated physicians, many participants stated that they learned to apply their pharmacology knowledge to real clinical situations only opportunistically during clinical rotations and the vast majority felt inadequately prepared for safe and effective usage of drugs [13]. Thus there might be a gap in transferring the knowledge acquired during medical school classes to real patient care.

Complex medical tasks like patient chart review and ward rounds are only seldom taught in medical school; students report performing them only sporadically during clerkships [14], [15]. In a study by de Feijter et al., medical students reported being torn between the acquisition of skills through practicing on real patients and delivering safe patient care, which led to an avoidance of performing tasks such as prescribing [16]. Medical students and novice graduates are only rarely supervised by experienced staff [17], [18].

Since young doctors have to perform ward rounds and patient chart review from day one of their graduation, medical education should address patient chart review and common patient hazards. In this study, we simulated the medical routine through complex patient management problems, thus we refer to the lessons as “problem-based training”. We did not use the checklist used in patient safety research but concentrated on common and easy to detect patient hazards instead.

Using a randomized, controlled cross-over-study, we investigated whether a structured problem-based patient chart review training improves the identification of common patient hazards, that is, diagnostic problems, medication problems, inadequate monitoring, and nosocomial infections, independent of context during patient chart reviews by advanced medical students.

## Methods

### Study Design and Participants

**Figure 1:**
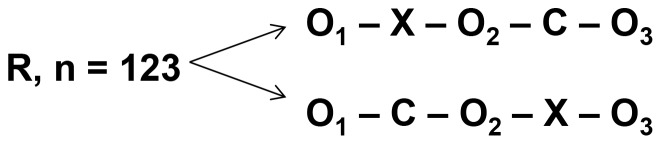
Study design. R = Randomization of 123 5^th^ year medical students, O = Observation (review of a fictional patient chart with twelve common patient hazards), X = Intervention (training on patient chart review and patient safety), C = Control-Intervention (ultrasound and Skills lab-training).

We conducted a prospective randomized trial with a cross-over design in order to identify potential superiority of the problem-based patient chart review training over the control intervention (see Figure 1). All 5^th^ year medical students participating in the mandatory internal medicine class (n = 123) were randomized into two equally large groups using a list of random numbers according to the following protocol: a list of five digit-random numbers was written down in three columns. Every student was assigned to one line, so that every student was pseudonymized with three random numbers, one for the first, one for the second and one for the third assessment. If the second digit of the first random number was odd, the student was assigned to the intervention – control group, if the second digit was even, he or she was assigned to the control – intervention group. For the rating, all assessment-patient charts were ordered according to the random numbers. The first rater rated them from top down, the second from bottom-up.

One group (intervention – control group, IC) received 15 hours of training in patient chart review and patient safety followed by a 15-hour control-intervention on abdominal ultrasound and skills-lab training. The other group (control – intervention, CI) received the control intervention first and the problem-based patient chart review training afterwards. While participation in the training was mandatory, the assessment was not. The students were free to decide, whether they submitted their assessment forms for the study or not. The students could withdraw from the study anytime without giving any reasons and without effect on their grading or other disadvantages. The medical students were blinded to the study question and the study design.

The study design is shown in figure 1. Before and after the interventions (problem-based patient chart review training, X, skills-lab training, C) the students were asked to review three fictional patient charts (observations, O_1–3_) in 30 minutes. The patient charts were standardized for twelve common patient hazards and permutated, so that every scenario served as first, second and third observation with the same frequency. The students were asked to address any problem they found in the patient chart but were not told that the focus of the assessment was on detection of patient hazards.

The students were asked to prepare for the main diagnoses (further details in the Assessment Section) three days in advance of each observation period, since we did not want to test their general knowledge about diseases. There were no changes in the study protocol after the trial commenced.

### Setting

The study was conducted at the University of Tübingen, Germany, a medical school with a traditional 6-year curriculum. The first two years are dedicated to basic sciences (e.g. anatomy, physiology, biochemistry). The core clinical medical training takes place during years 3–5, the 6^th^ (final) year comprises three four-month internships in internal medicine, surgery and an elective. Our students were all in the fifth year, so they completed most of their clinical training (pharmacology classes, lessons on internal medicine, etc.).

### Assessment

Patient management problems have a good face validity and have been used successfully for teaching and assessment [19]. The actual patient hazard patterns used in this study were based on recent publications on patient safety [2], [6], [20], [21], [22].

We thus prepared fictional patient charts with three different scenarios (cellulitis in an immunosuppressed patient, atrial fibrillation in hyperthyroidism, patient with advanced cancer in pain) and standardized the patient charts with twelve common patient hazards:

One indicated medication missing
One medication not indicated
One medication with the wrong dosage
One risk situation for a non-authorized medication
One side effect of the medication
One contraindication against a medication
One incidental diagnostic finding
A missing diagnostic tests for the main problem
Missing monitoring
One infectious complication
Wrong diet/fluid management
Incomplete documentation

Table 1 shows an exemplary case vignette as used in the study.

**Table 1:** Example of a case vignette with twelve common patient hazards.

Scenario	Cellulitis in patient with a renal graft, penicillin allergy
Drug missing	Thromboembolic prophylaxis
Not-indicated medication	Penicillin
Medication with wrong dosage	Immunosuppression 10-fold overdosed
contraindication against a drug	Penicillin allergy
Side effect of the medication	Steroid diabetes
Risk situation for non-authorized medication	Pain killer
Incidental finding	Atypical naevus
Missing diagnostic test for the main problem	Blood culture
Missing monitoring	Tacrolimus-level
Infectious complication	Not used central line catheter
Wrong Diet/fluid management	Exsiccosis
Incomplete Documentation	Anemia

The actual patient hazards were different in the three scenarios. The scenarios were developed by two physicians and approved for face validity by another four physicians. We constructed the fictional patient charts so that they would resemble the real patient charts used in our tertiary care hospital. They comprised: a cover sheet with patient data, the history, main diagnosis and comorbidities and results of the main examinations, the actual patient chart recording the patient’s vital signs, the medication, nursing notes and the laboratory results.

The students were asked to review the patient chart and amend documentation, prescribe medication, and order diagnostic tests as they saw fit for any problem they identified. We only assessed, whether the problem was identified, not whether subsequent measures were appropriate. The prescription and documentation forms were subsequently rated by two blinded raters using a checklist for every problem addressed. The scores were calculated as number of problems found divided through total number of problems in the patient chart.

### Intervention

According to Kolb and Fry, an effective training comprises four steps: concrete experience, observation and reflection, the formation of abstract concepts, and testing in new situations [23]. This was the conceptual framework for the patient chart review-training. The first assessment on patient chart review, which was done before any intervention, served as the concrete experience. Reflection and formation of abstract concepts was supposed to be facilitated by the theoretical input and discussion of the cases during the patient chart review training. The assessment in a different context after the problem-based patient chart review training (O_2_ and O_3_ for the IC, O_3_ for the CI) served as the testing in new situations.

The patient chart review-training lasted one week and comprised:

A patient chart reading exercise in small groups (90 minutes)
A lecture on common patient hazards (45 minutes)
Preparation of seminar papers on various topics concerning patient management (e.g. indications for thromboembolic prophylaxis, optional medication)
Three group exercises to prepare standardized fictional patient charts containing common patient hazards in small groups (90 minutes each)
Three lectures discussing the prepared standardized fictional patient charts with a lecturer (90 minutes each)

The control-intervention also lasted one week and comprised training in the skills-lab on abdominal ultrasound (8 h) and puncture-techniques (central line catheter, pleura, arterial puncture, bone marrow) on a manikin (7 h).

### Statistics

We used JMP 9.0 (SAS Institute Inc, Cary, NC, USA) to compute the results. Calculations were based on mean rater evaluations. Since the values of the identified patient hazards were skewed, they were compared using the Wilcoxon rank-sum test and displayed as mean and interquartile ratio (IQR). We performed a power analysis (G*Power software, Erdfelder, Faul, & Buchner, 1996, Düsseldorf, Germany) based on a previous investigation on prescription errors [24].

Our null hypothesis was defined as no difference between the percentage of recognized patient hazards after the problem-based intervention compared to the control-intervention.

### Ethics

The study was conducted in accordance with the Declaration of Helsinki (revised form, Seoul 2008). The study protocol was approved by the local Ethics Committee, decision number 488/2010A. The medical students gave written consent. Study participation was voluntary; the students could withdraw without giving any reason. No harms or adverse effects were encountered.

## Results

According to the power analysis we could detect a difference of 10% with n = 74, α = .05 and SD =  .15.

The overall interrater-reliability was .96.

We had to exclude 35 students due to missing data since they did not complete all three assessments. The characteristics of the remaining 88 students are shown in Table 2.

**Table 2:** Characteristics of the study participants.

	IC	CI
Age	26±3 years	26±3 years
Gender	13 male, 30 female	23 male, 22 female
Previous education	2 nurse/paramedic, 5 aide on the ward, 36 none	4 nurse/paramedic, 5 aide on the ward, 36 none
Previously performed patient chart reviews	40 never, 3 1–5 times	40 never, 3 1–5 times, 2>5 times
N	43	45

Overall, the students identified 17% (IQR 8–29%) of the patient hazards at O_1_ 56% (IQR 41–66%) of the patient hazards at O_3_, p<.0001.

**Figure 2:**
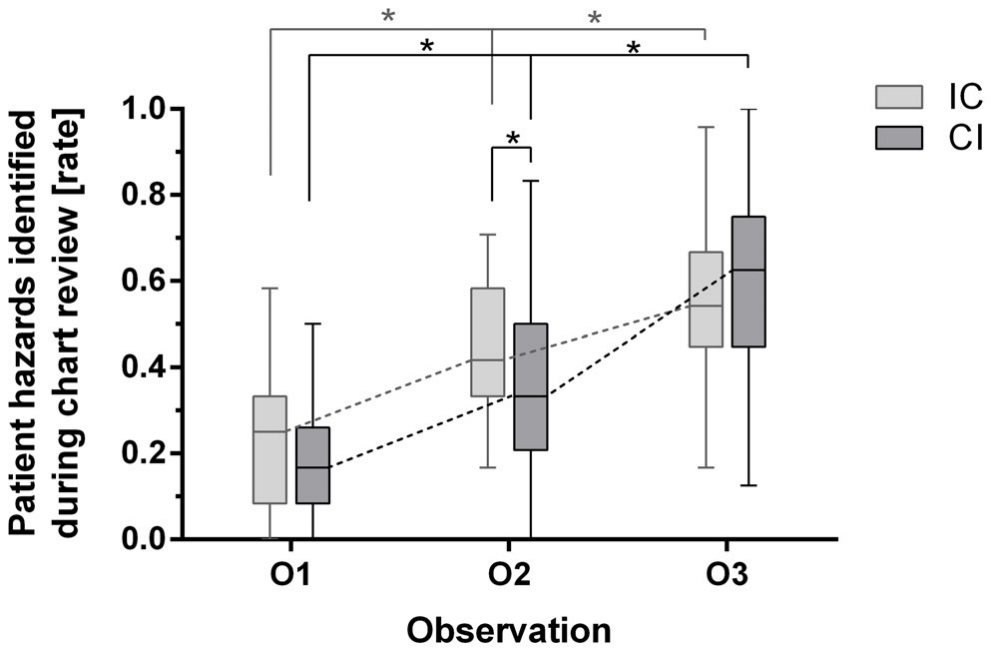
Patient hazards identified during patient chart review. IC: intervention – control-group (first patient chart review-training, then control-intervention), CI: control – intervention-group (first control-intervention, then patient chart review-training). O_1–3_: observations, review of a standardized fictional patient chart with twelve common patient hazards. A star indicates a significant difference (all p<.01).

At O_1_, when no group had patient chart review training, the scores of the groups did not differ (IC mean 21% (IQR 8–33%) vs. CI mean 17% (IQR 8–21%), p = .95). At O_2_, when the IC already had patient chart review-training but the CI not, the scores differed significantly (IC mean 42% (IQR 33–58%) vs. CI mean 29% (IQR 21–42%), p<.0001). At O_3_, when both groups had the problem-based patient chart review training, there was again no significant difference (IC mean 54% (IQR 43–66%) vs. CI mean 63% (IQR 42–72%), p = .26). At O_2_ the students identified significantly more patient hazard than during O_1_ and even more during O_3_. The effect was more pronounced after problem-based patient chart review training but also significant after the control intervention, although the assessments comprised different scenarios (all p<.01) (see Figure 2).

## Discussion

In our study, the skill of advanced medical students to identify patient hazards during patient chart review improved after problem-based patient chart review training. The effect was partly context-independent, so it was possible to train the students on a case with advanced cancer and the students were able to apply the skill to a case with atrial fibrillation and hyperthyroidism. The main reason for this phenomenon might be, that we taught the students to look for patterns that may impose patient hazards, and most of these patters do not necessarily require context-specific knowledge (i.e. check every drug for adequate dosing) or refer to knowledge already covered during previous classes (e.g. drug-drug-interactions taught in their pharmacology classes). In addition, the raised awareness for patient safety may have had an influence on the way the students review the patient charts.

Moreover, the students improved most when they had the problem-based patient chart review training, but also identified more patient hazards after the control intervention only.

This corresponds with a previous study on prescription errors, where the ability to critically appraise medication and to modify it to a specific clinical context was improved independent of context with a problem-based training [25]. In a previous study we investigated whether clerkships or previous training improve the identification of patient hazards during patient chart review and found that clerkships alone did not have an effect, while students, who reported to have performed at least one patient chart review themselves, identified significantly more patient hazards than students who had never performed a patient chart review [24]. So the improvement after the control-intervention might actually be an improvement due to the practice in patient chart review during the observations. While the students might be able to solve the tasks when presented with bare facts, finding the patterns in a patient chart bursting with information constitutes a much bigger challenge, which reflects the daily work of physicians in a more realistic manner.

Our study has several limitations. Since study participation was voluntary and the interventions and assessments lasted two weeks, we had to exclude 28% of the students due to missing data, so there might be a distortion in the study sample. The students only reviewed fictional standardized patient charts, not real patient charts. While we tried to balance the patient hazards carefully, the students might have stopped looking for problems after identifying several patient hazards, since they did not expect so many patient hazards in one patient chart. In addition, we cannot exclude that the students IC group discussed the problem-based patient chart review training with the CI-group at any point, although we asked them not to.

While the medical students were blinded to the study question and the study design, the instructor was not. The identification is only a procedural outcome and it has yet to be proven that the identification of patient hazards can actually avert patient harm. We cannot exclude that a simple lesson on patient safety alone might also have improved the ability to identify patient hazards, since the students received this lesson during the patient chart review training.

Further research is warranted on the ability of medical students and novice graduates to read patient charts and to identify patient hazards and whether the training has an effect on patient safety.

In conclusion, a problem-based patient chart review training and patient chart review practice together improve the skills of advanced medical students in identifying patient hazards during patient chart review and thus might probably contribute to patient safety.

## References

[1] Chapman NE (2001) From behind closed doors. Healthc Inform 18: 37–40, 42. 11727659

[2] de Vries EN, Ramrattan MA, Smorenburg SM, Gouma DJ, Boermeester MA (2008) The incidence and nature of in-hospital adverse events: a systematic review. Qual Saf Health Care 17: 216–223. 18519629 10.1136/qshc.2007.023622 PMC2569153

[3] Rancourt C, Moisan J, Baillargeon L, Verreault R, Laurin D, et al (2004) Potentially inappropriate prescriptions for older patients in long-term care. BMC Geriatr 4: 9. 15488143 10.1186/1471-2318-4-9 PMC529256

[4] Lesar TS, Briceland L, Stein DS (1997) Factors related to errors in medication prescribing. Jama 277: 312–317. 9002494

[5] Lesar TS, Lomaestro BM, Pohl H (1997) Medication-prescribing errors in a teaching hospital. A 9-year experience. Arch Intern Med 157: 1569–1576. 9236558

[6] Krahenbuhl-Melcher A, Schlienger R, Lampert M, Haschke M, Drewe J, et al (2007) Drug-related problems in hospitals: a review of the recent literature. Drug Saf 30: 379–407. 17472418 10.2165/00002018-200730050-00003

[7] Callen J, Georgiou A, Li J, Westbrook JI (2011) The safety implications of missed test results for hospitalised patients: a systematic review. BMJ Qual Saf 20: 194–199. 10.1136/bmjqs.2010.044339 PMC3038104 21300992

[8] Zwaan L, de Bruijne M, Wagner C, Thijs A, Smits M, et al (2010) Patient record review of the incidence, consequences, and causes of diagnostic adverse events. Arch Intern Med 170: 1015–1021. 20585065 10.1001/archinternmed.2010.146

[9] Murff HJ, Patel VL, Hripcsak G, Bates DW (2003) Detecting adverse events for patient safety research: a review of current methodologies. J Biomed Inform 36: 131–143. 14552854 10.1016/j.jbi.2003.08.003

[10] de Feijter JM, de Grave WS, Muijtjens AM, Scherpbier AJ, Koopmans RP (2012) A comprehensive overview of medical error in hospitals using incident-reporting systems, patient complaints and chart review of inpatient deaths. PLoS One 7: e31125. 22359567 10.1371/journal.pone.0031125 PMC3281055

[11] Nikendei C, Kraus B, Schrauth M, Briem S, Junger J (2008) Ward rounds: how prepared are future doctors? Med Teach 30: 88–91. 18278658 10.1080/01421590701753468

[12] Celebi N, Wagner R, Weyrich P, Heine D, Fenik Y, et al (2012) Clerkships do not improve recognition of patient hazards by advanced medical students during chart review. Med Teach 34: 1087. 10.3109/0142159X.2012.716570 22931111

[13] Heaton A, Webb DJ, Maxwell SR (2008) Undergraduate preparation for prescribing: the views of 2413 UK medical students and recent graduates. Br J Clin Pharmacol 66: 128–134. 18492128 10.1111/j.1365-2125.2008.03197.x PMC2485268

[14] Remmen R, Derese A, Scherpbier A, Denekens J, Hermann I, et al (1999) Can medical schools rely on clerkships to train students in basic clinical skills? Med Educ 33: 600–605. 10447847 10.1046/j.1365-2923.1999.00467.x

[15] Celebi N, Tsouraki R, Engel C, Holderried F, Riessen R, et al (2012) Does doctors’ workload impact supervision and ward activities of final-year students? A prospective study. BMC Med Educ 12: 24. 22540897 10.1186/1472-6920-12-24 PMC3372449

[16] de Feijter JM, de Grave WS, Dornan T, Koopmans RP, Scherpbier AJ (2011) Students’ perceptions of patient safety during the transition from undergraduate to postgraduate training: an activity theory analysis. Adv Health Sci Educ Theory Pract 16: 347–358. 21132361 10.1007/s10459-010-9266-z PMC3139877

[17] Howley LD, Wilson WG (2004) Direct observation of students during clerkship rotations: a multiyear descriptive study. Acad Med 79: 276–280. 14985204 10.1097/00001888-200403000-00017

[18] Grant J, Kilminster S, Jolly B, Cottrell D (2003) Clinical supervision of SpRs: where does it happen, when does it happen and is it effective? Specialist registrars. Med Educ 37: 140–148. 12558885 10.1046/j.1365-2923.2003.01415.x

[19] Marquis Y, Chaoulli J, Bordage G, Chabot JM, Leclere H (1984) Patient-management problems as a learning tool for the continuing medical education of general practitioners. Med Educ 18: 117–124. 6700446 10.1111/j.1365-2923.1984.tb00984.x

[20] Vincent C (2010) Patient Safety: Wiley-Blackwell.

[21] Kripalani S, LeFevre F, Phillips CO, Williams MV, Basaviah P, et al (2007) Deficits in communication and information transfer between hospital-based and primary care physicians: implications for patient safety and continuity of care. Jama 297: 831–841. 17327525 10.1001/jama.297.8.831

[22] Vogt EM (2002) Effective communication of drug safety information to patients and the public: a new look. Drug Saf 25: 313–321. 12020171 10.2165/00002018-200225050-00002

[23] Kolb DA FR (1975) Toward an applied theory of experiential learning; Cooper C, editor. London: John Wiley.

[24] Celebi N, Kirchhoff K, Lammerding-Koppel M, Riessen R, Weyrich P (2010) Medical clerkships do not reduce common prescription errors among medical students. Naunyn Schmiedebergs Arch Pharmacol 382: 171–176. 20535451 10.1007/s00210-010-0530-9

[25] Celebi N, Weyrich P, Riessen R, Kirchhoff K, Lammerding-Koppel M (2009) Problem-based training for medical students reduces common prescription errors: a randomised controlled trial. Med Educ 43: 1010–1018. 19769651 10.1111/j.1365-2923.2009.03452.x

